# Association of atmospheric temperature with out-of-hospital natural deaths occurrence before and during the COVID-19 pandemic in Osaka, Japan

**DOI:** 10.1038/s41598-023-45816-7

**Published:** 2023-10-28

**Authors:** Hidenori Yoshizawa, Satoshi Hattori, Ken-ichi Yoshida, Hideyuki Maeda, Tetsuhisa Kitamura, Eiichi Morii

**Affiliations:** 1 Department of Diagnostic Pathology, Osaka University Hospital, Suita City, Osaka Japan; 2 Osaka Prefectural Office of Medical Examiner, 1-6 Bamba-cho, Chuo-ku, Osaka City, 540-0007 Osaka Japan; 3 Department of Biomedical Statistics, Graduate School of Medicine, Osaka University, Suita City, Osaka Japan; 4 Department of Legal Medicine, Graduate School of Medicine, Osaka University, Suita City, Osaka Japan; 5 Department of Social and Environmental Medicine, Graduate School of Medicine, Osaka University, Suita City, Osaka Japan; 6 Department of Pathology, Graduate School of Medicine, Osaka University, Suita City, Osaka Japan

**Keywords:** Epidemiology, Public health

## Abstract

In this study, we aimed to investigate the relationship between out-of-hospital natural death (OHND) and ambient temperature and examine the seriousness of the impact of the coronavirus disease-2019 (COVID-19) pandemic on this relationship. We used data from the Osaka Prefectural Office of Medical Examiners between 2018 and 2022 and performed a retrospective observational study. A Poisson regression model was applied to examine the relationship between OHND and temperature in Osaka City. The relative risk of OHND at 5 °C and 32 °C compared to the minimum mortality temperature increased from 1.81 in the pre-COVID-19 period to 2.03 in the post-COVID-19 period at 5 °C and from 1.29 in the pre-COVID-19 period to 1.60 in the post-COVID-19 period at 32 °C. The increase in relative risk per 1 °C increase from the pre- to post-COVID-19 period was 1.0551 (rate ratio [RR], *p* = 0.003) in the hot environment and 1.0233 (RR, *p* = 0.013) in the cold environment, which was larger than that in the hot environment. Although the risk of OHND increased at both temperatures, the change in OHND risk during post-COVID-19 was larger in the hot environment than in the cold environment, implicating the effect of pandemics in the current scenario of global warming.

## Introduction

In Japan, unnatural deaths undergo a death investigation and cause of death estimation by medical examiners (MEs) after the exclusion of criminal cases in some special districts, including Osaka. Cases of solitary death at home or unexpected out-of-hospital death are temporarily treated as unnatural deaths; however, in special districts with an ME system, ME examinations determine these as out-of-hospital natural deaths (OHND). OHND is an important epidemiological issue considering that it includes many solitary and out-of-hospital sudden deaths; epidemiologic investigation of OHND is also consistent with the essential purpose of the ME system.

Temperature-related mortality has been a serious public health concern in association with the worldwide anathermal climate. High and low temperatures have been implicated in all-cause mortality^1,2^, sudden cardiac death^3^, all-natural causes of death, and major cardiopulmonary diseases^4^. Meanwhile, the risk of out-of-hospital cardiac arrest (OHCA) has been shown to increase with extremely high or low temperatures^5–7^. However, all of these studies are based on pre-coronavirus disease-2019 (pre-COVID-19) data, and no studies have yet compared the relationship of ambient temperature with either mortality or OHCA risk using post-COVID-19 data.

In addition, the risk of OHCA has been shown to increase owing to the COVID-19 pandemic^8,9^. Marijon et al. reported in their multi-center cohort study that the incidence of non-traumatic OHCA at home increased in association with less frequent bystander cardiopulmonary resuscitation and much delayed intervention during COVID-19 in Paris and its suburbs; they suggested that the results were probably indirect effects associated with COVID-19-related behavioral restrictions and adjustment of health-care services^10^. In Japan, the Fire and Disaster Management Agency investigated and reported the tightness of the emergency medical system (EMS) during COVID-19 (https://www.fdma.go.jp/disaster/coronavirus/post-1.html).

Thus, OHND, which includes many solitary and out-of-hospital sudden deaths, might be more affected by COVID-19 than OHCA and might be more closely associated with temperature changes compared to the pre-COVID-19 period. Based on this hypothesis, we investigated the effect of COVID-19 on the relationship between OHND and temperature using OHND data from the Osaka Prefectural Office of ME in this study. In addition, subgroup analyses were performed, stratified by the relevant factors reported in previous studies on OHCA^11,12^.

## Results

From 2018 to 2022 at the Osaka Prefectural Medical Examiner's Office, there were 16,872 cases classified as deaths due to internal causes, excluding bathing-related deaths. Of these, 7115 cases were from January 2018 to March 2020, considered as the pre-COVID-19 time, and 9757 cases were from April 2020 to December 2022, considered as the post-COVID-19 time. In Osaka City, the weekly mean temperatures ranged from 2.7 to 32.3 °C, with a median of 18.1 °C; the first quartile temperature was 10.8 °C and that of the third quartile was 25.1 °C during the study period.

**Figure 1:**
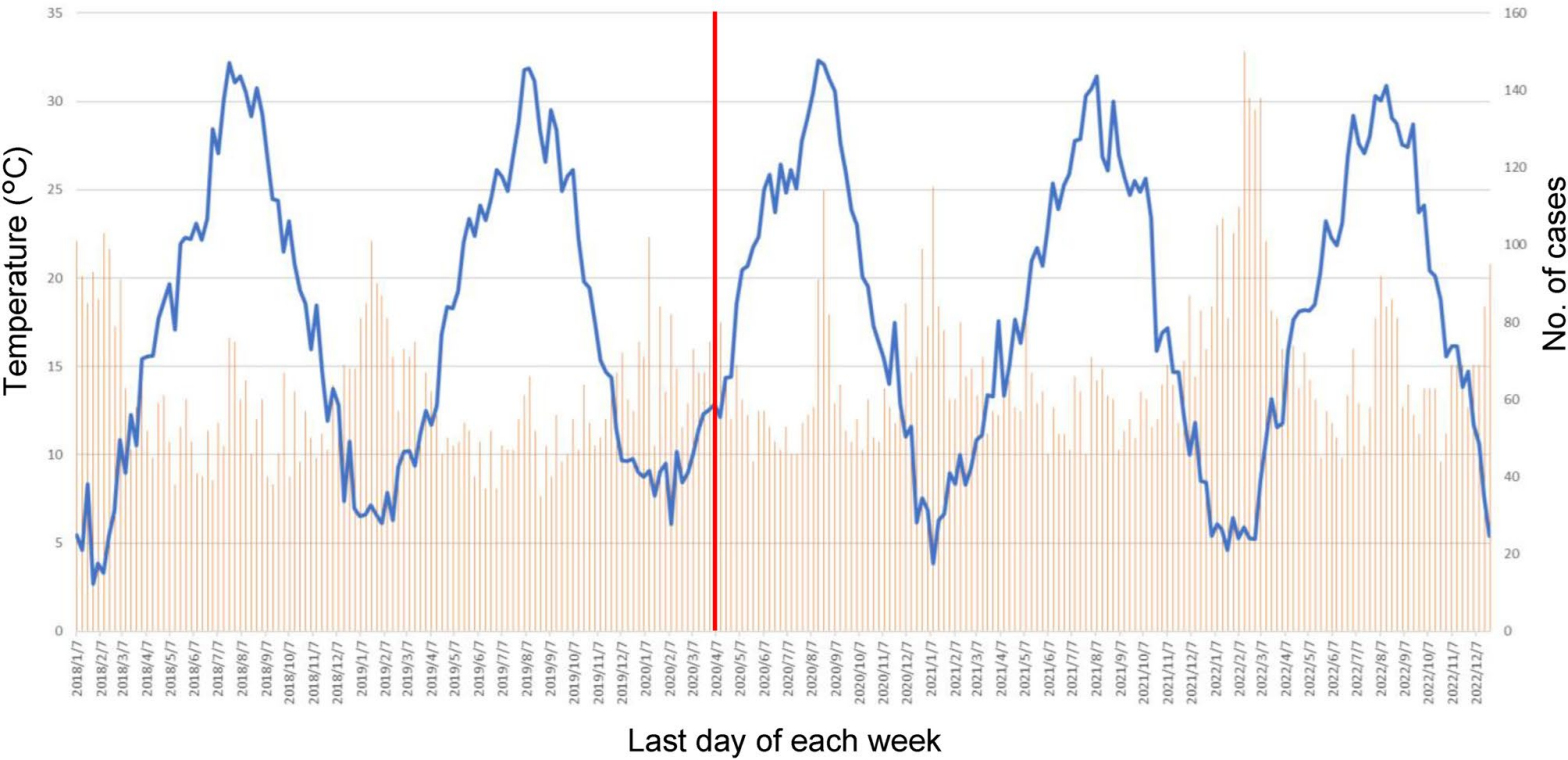
The weekly mean temperature and the weekly total number of cases. The weekly mean temperature is shown in the blue line graph. The total weekly number of cases is shown in the orange bar chart. The left axis shows the temperature and the right axis shows the number of cases. While the up and down temperature patterns reflect seasonal changes, the bar chart shows that the number of cases also shows a cyclical pattern of fluctuation. The timing of the increase in cases shows synchronization with both the timing of the temperature rise and fall. This figure vaguely captures the increasing trend in the number of cases post-COVID-19 pandemic, however the change in the relationship with temperature is ambiguous.

**Figure 2:**
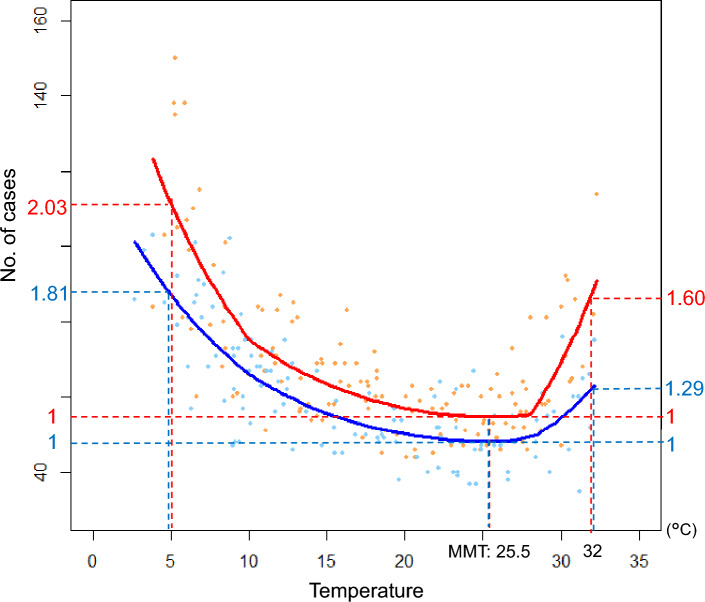
Scatterplot of the relationship between temperature and the number of cases. The relationship between temperature and the number of cases is shown with a scatterplot, and the relationship between temperature and predictive values obtained from the regression model is overlaid on the figure as regression curves. The minimum mortality temperature (MMT) and the relative mortality risk to MMT are also listed on the horizontal and bilateral vertical axes in the figure. The relative risk of mortality is calculated as the ratio of the number of cases at 5 °C and 32 °C versus at MMT, and the pre-COVID-19 period is noted in blue and the post-COVID-19 period in red. The number of cases and regression curves are color-coded pre- and post-COVID-19 period. The number of cases in the pre- and post-COVID-19 period is shown by light blue and orange dots, and the regression curves of pre- and post-COVID-19 periods by blue and red lines, respectively. This figure indicates that the timing of the increase in the cases coincides with the timing of both the temperature rise and fall, as shown in Fig. 1. The regression curves show J-shapes owing to non-linear and additional local linear relationships. At temperatures colder than MMT (25.5 °C), cases increase gradually; at hotter temperatures, cases increase rapidly.

Figure 1 shows the weekly mean temperature and the weekly total number of cases, with the temperatures connected by line graphs and the number of cases by a bar chart. The results of the Poisson regression analysis are shown in Fig. 2 and Table 1.

**Table 1:** Association of temperature, COVID-19 period, and their interactions on the mortality risk.

	RR (95% CI)	*p*-value
Overall relationship		
With T: exp (β_1_)	0.9349 (0.9117–0.9586)	< 0.001
With T^2^: exp (β_2_)	1.0013 (1.0007–1.0020)	< 0.001
Relative risk of post- vs pre-COVID-19 period: exp (γ)		
	1.1391 (1.0969–1.1829)	< 0.001
Common effects of pre- and post-COVID-19 period		
Relative risk per 1 °C in hot environment (T > 28 °C): exp (δ_1_)	1.0508 (1.0100–1.0932)	0.014
Relative risk per 1 °C in cold environment (T < 10 °C): exp (η_1_)	1.0076 (0.9821–1.0338)	0.563
Impacts of COVID-19 pandemic on		
Relative risk per 1 °C in hot environment (T > 28 °C): exp (δ_2_)	1.0551 (1.0189–1.0927)	0.003
Relative risk per 1 °C in cold environment (T < 10 °C): exp (η_2_)	1.0233 (1.0048–1.0421)	0.013

CI indicates the confidence interval, and RR is the rate ratio.

In the table, the estimated rate ratios (RRs), which are exponentially transformed from the respective regression coefficients (exp(.)), are listed.

The association between temperature and the risk of OHND cannot be directly interpreted from β_1_ and β_2_ because of the non-linear relationship. Therefore, the overall relationship with temperature should be interpreted using Fig. 2

RR, exp (γ), represents the relative risk of the post- to pre-COVID-19 period across the overall temperature range. Although δ_1_ and η_1_ indicate the local linear effects added to the non-linear relationship, which provide better fitting to the regression model commonly pre- and post-COVID-19 periods, these are not essential for interpreting the results of the analysis.

The RR, exp (δ_2_), represents how the relative risk per 1 °C changed post- versus pre-COVID-19 pandemic in the hot environment (T > 28 °C), and also represents, in the figure, the change in gradient of the regression curve post- to pre-COVID-19 period in the high-temperature region. Similarly, RR, exp (η_2_), represents how the relative risk per 1 °C in the cold environment (T < 10 °C) changed and the change in gradient of the regression curve in the cold temperature region.

Intercept term, exp (a), is omitted from the list as it is not necessary for interpreting the results.

Table 1 shows the effects of the factors based on the estimated values for the respective regression coefficients. In Table 1, the row for the *“Overall relationship”* shows the baseline relationship between the number of OHND cases and the mean temperature. The quadratic term is highly significant, and a non-linear relationship is suggested. From the results in the row for the “*Relative risk in post- vs. pre-COVID-19 periods*,” the number of cases in the post-COVID-19 period is 1.1391 (95% confidence interval [CI]: 1.0969–1.1829, *p* < 0.001) times higher than those in the pre-COVID-19 period. The row for the *“Effect of the COVID-19 pandemic”* evaluates the effect of COVID-19 specific to cold and hot environments. The increase in relative risk per 1 °C in the post-COVID-19 period compared to that in the pre-COVID-19 period is 1.0551 (rate ratio [RR], *p* = 0.003) in the hot environment (T > 28 °C) and 1.0233 (*p* = 0.013) in the cold environment (T < 10 °C), which is larger in the hot environment.

The scatterplots in Fig. 2 show the relationship between temperature and the number of cases and the expected number of cases based on the result of Poisson regression analysis. It indicates that the fitted Poisson regression captures the actual case counts and fits well (See also Supplementary Fig. S1 online). The significant effects shown in Table 1 are evident in Fig. 2, where the curve indicating the overall number of cases of the post-COVID-19 period shifts upward compared to the pre-COVID-19 period. In addition, the gradient of the curve also shows a steeper decrease around lower temperatures and an even steeper increase around higher temperatures in the post-COVID-19 period than in the pre-COVID-19 period. To represent these significant effects of interaction terms in a more intuitive way, we set the temperature providing the minimum predictive case number (minimum mortality temperature [MMT]) to 25.5 °C, which was estimated by Poisson regression, as a reference, and the extremely hot and cold temperatures were set to 32 °C and 5 °C, respectively, which are the approximate maximum and minimum temperatures in the observed overall temperature. In the post-COVID-19 period compared to that in the pre-COVID-19 period, the relative risk referenced to MMT increased from 1.81 to 2.03 at extremely cold temperature, which corresponded to the change in the absolute value differential in the number of events from 39.0 to 56.6, and from 1.29 to 1.60 at extremely hot temperature, which was from 14.0 to 32.9 in the absolute value differential. However, the increase in gradients in the post-COVID-19 period compared to the pre-COVID-19 period was greater in the high-temperature region (> 28 °C) than in the low-temperature region (< 10 °C), as shown by relative risk per 1 °C for the *“Effect of the COVID-19 pandemic”* in Table 1.

The sensitivity analysis results were similar to those of the main analysis (Supplementary Table S1,2 online).

**Figure 3:**
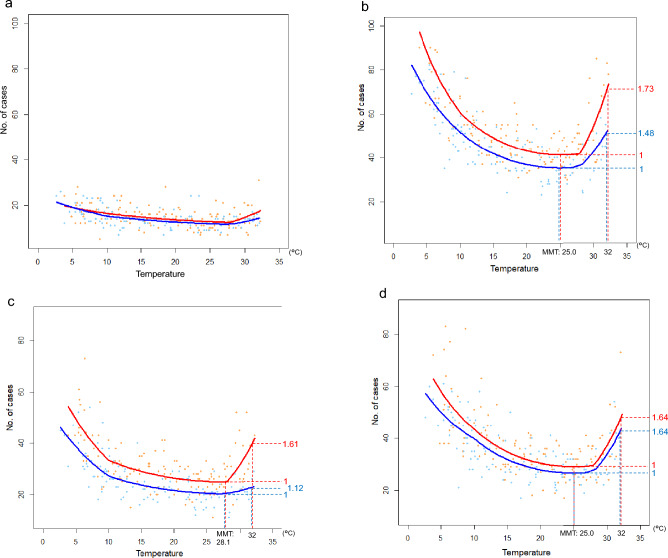
Subgroup analysis stratified by age and living style. (**a**), (**b**) show the results of subgroup analysis stratified by age. Color-coded pre- and post-COVID-19 as in Fig. 2. (**a**) Non-elderly group. This figure shows that the non-elderly group was not affected by either the COVID-19 pandemic or the temperatures. (**b**) Elderly group. In the elderly group, the number of cases across the overall temperature range increased post-COVID-19. The elderly were affected by the hot environment in both the pre- and post-COVID-19 periods, unlike the non-elderly. However, the relative risk at 32 °C increased from 1.48 in the pre-COVID-19 period to 1.73 in the post-COVID-19 period, and the gradient of the high-temperature region is steeper in the post- than in the pre-COVID-19 period, indicating that the influence of hot environment is greater in the post-COVID-19 period. (**c**), (**d**) show the result of subgroup analysis stratified by living alone or not. Color-coded pre- and post-COVID-19 as in Fig. 2. (**c**) Living with a housemate group. In the living with housemate group, the number of cases across the overall temperature range increased post-COVID-19. Pre-COVID-19 pandemic, the relative risk at 32 °C is 1.12, and the gradient of the high-temperature region is slightly steep. Meanwhile, post the COVID-19 pandemic, the relative risk at 32 °C is greatly increased to 1.61, and the gradient of the high-temperature region is sharply steeper. (**d**) Living alone group. In the living-alone group, the relative risk at 32 °C did not change during the post-COVID-19 period compared to the pre-COVID-19 period. Notably, it appears that the number of cases increased across the overall temperature range in the post-COVID-19 period. This is due to an increase in the population living alone after the post-COVID-19 period. The model, in which the change in the living alone population is included as the offset term, indicates that the relative risk of the living alone group significantly reduced across the overall temperature range in the post-COVID-19, as shown in Table S2.

As a result of stratification, the elderly group contained 13,042 (77.3%) individuals and the living-alone group contained 9444 (56.0%), as shown in Fig. 3a–d (Supplementary Table S3 online).

As shown in Fig. 3a, the non-elderly group was not affected by temperature or the COVID-19 pandemic. As shown in Fig. 3b, in the elderly group, there was an increase in the number of OHND cases in the post-COVID-19 period across the overall temperature range; indeed, the relative risk of the post- versus pre-COVID-19 period was significant (RR: 1.1565 [*p* < 0.001]) (Supplementary Table S3 online). The relative risk at the extremely hot temperature increased from 1.48 in the pre-COVID-19 period to 1.73 in the post-COVID-19 period, and the gradient of the regression curve in the high-temperature range was steeper in the post- than in the pre-COVID-19 period. Indeed, the increase in relative risk per 1 °C from pre- to post-COVID-19 period was not significant (RR:1.0155 [*p* = 0.145]) in the cold environment (T < 10 °C), whereas it was significant (RR:1.0403 [*p* = 0.042]) in the hot environment (T > 28 °C) (Supplementary Table S3 online). The sensitivity analysis results with a reference age of 75 years showed a trend similar to that of the main subgroup analysis (Supplementary Table S4 online).

As shown in Fig. 3c, in the living with a housemate group, the number of OHND cases increased in the post-COVID-19 period across the overall temperature range; indeed, the relative risk of the post- versus pre-COVID-19 period was significant (RR: 1.2659 [*p* < 0.001]) and the largest among all subgroups. The relative risk at the extremely hot temperature increased from 1.12 in the pre-COVID-19 period to 1.61 in the post-COVID-19 period. The increase in relative risk per 1 °C from the pre- to post-COVID-19 period was significant only in the hot environment (T > 28 °C) (RR:1.0947 [*p* = 0.001]) (Table S3). Conversely, as shown in Fig. 3d, in the living-alone group, the relative risk at the extremely hot temperature was as high as 1.64 in the pre-COVID-19 period and remained the same in the post-COVID-19 period. The change in relative risk per 1 °C in the pre- and post-COVID-19 periods was insignificant in the hot environment (Supplementary Table S3 online) as well as in the cold environment (T < 10 °C) in all subgroups.

## Discussion

This analysis of out-of-hospital natural death (OHND) at the Osaka Prefectural Medical Examiner’s Office showed an increased risk of OHND due to hot and cold environments that was observed among the elderly and the living-alone groups in this study population. Compared to the pre- and post-COVID-19 periods, the risk of OHND for these two groups increased only in the hot environment. These results provide insight into the risk of OHND worldwide during pandemics such as COVID-19 or in the current scenario of global warming.

The risk of OHND in a hot environment increased in the post-COVID-19 time. Whereas the risk of OHND in the cold environment increased only marginally in the post-COVID-19 period, that in the hot environment increased remarkably. Thus, the effect of the COVID-19 pandemic increased the risk of OHND predominantly in hot environments than in cold environments. This suggests that the risk of OHND will be further increased by COVID-19 and the effects of global warming.

The risk of OHND increased in the post-COVID-19 time across the overall temperature range and that of OHND in hot environments further intensified in the elderly group. A previous study on OHCA reported that the risk of OHCA was related to the proportion of older individuals in a population^11^; moreover, older individuals were significantly more prone to OHCA in a hot environment^7^ and during COVID-19^8^. In the current study targeting OHND, the elderly group was affected by COVID-19 as well as the hot environment, whereas the non-elderly group was unaffected by either COVID-19 or temperature. Furthermore, among the elderly group, the trend toward increased risk of OHND with higher temperatures intensified in the post-COVID-19 time. Therefore, in the post-COVID-19 time, the elderly should be considered a high-risk group for OHND in a hot environment. Japan has a super aging population greater of the rest of the world, and the findings from this study revealed the risk of OHND incidence in the elderly in high temperature environments. In the future, other industrialized countries are expected to show similar trends as their society ages; therefore, we should focus on the trends of increased OHND risk worldwide.

The risk of OHND intensified in the hot environment in the post-COVID-19 time in the living with a housemate group. In contrast, the risk was higher even in the pre-COVID-19 period in the hot environment but remained unchanged in the post-COVID-19 period in the living-alone group. In the pre-COVID-19 period, the risk of OHND in the hot environment was higher in the living-alone group, whereas it was lower in the living with a housemate group (the relative risk of the 32 °C versus MMT was 1.64–1.12). This result might suggest that social isolation among the living-alone group affects the risk of OHND in a hot environment.

In the post-COVID-19 period, the trend toward increased risk of OHND with higher temperatures significantly intensified among the living with a housemate group (RR 1.0947, *p* = 0.001). Comparing the relative risk of the 99th percentile temperature versus MMT in the post-COVID-19 period, it was almost equal among the living-alone and living with a housemate groups (1.61 and 1.64, respectively). Considering the relationship between social isolation and the risk of OHND in the hot environment, as mentioned above, this result indicates that the social isolation of the living with a housemate group was exacerbated in the post-COVID-19 period and progressed to the same degree as that of the living-alone group. Meanwhile, among the living-alone group, the risk of OHND in the hot environment was almost the same in the pre- and post-COVID-19 periods, indicating that the social isolation of the living-alone group was little affected by COVID-19. In the post-COVID-19 world, the risk of OHND in a hot environment in the living with a housemate group should not be underestimated.

To the best of our knowledge, we report for the first time that the risk of OHND increased across the entire temperature range in the post-COVID-19 time among the living with a housemate group. The number of cases of OHND in the living-alone group increased slightly post-COVID-19 across the overall temperature range, as shown in Fig. 3d. However, in the evaluation of the relative risk in the post- versus pre-COVID-19 period, the increased number of people living alone in Osaka City should be considered, as mentioned in the methods section. In the model with offset terms of the population of the living-alone or living with a housemate group, the relative risk of the living alone group in the post- versus pre-COVID-19 period was significantly lower than 1 (RR: 0.9142, *p* = 0.001), whereas, in the living with a housemate group, it was larger than 1 (RR: 1.2659, *p* < 0.001) (Supplementary Table S3 online). A previous study on OHCA and a comparison of pre- and post-COVID-19 times reported that the risk of OHCA was related to the proportion of households with no family nucleus^11^, and that the episodes of unwitnessed cardiac arrest increased during COVID-19^12^. People living alone were also speculated to have been at a higher risk of unwitnessed cardiac events, which are associated with poorer outcomes, owing to the stay-at-home order during the COVID-19 pandemic^13^. In this analysis targeting OHND, in this population, the risk of OHND across the overall temperature range increased in the post-COVID-19 period not in the living-alone group but in the living with a housemate group.

Changes in the trends in the risk of OHND among the living with a housemate group should be monitored closely in the future.

The results of this study demonstrate an increased risk of OHND during the COVID-19 pandemic, which suggests that the risk of OHND is influenced by limited access to healthcare, changes in individual behavior patterns, and mental stress levels resulting from the COVID-19 pandemic. In particular, during the COVID-19 pandemic, the shortage of medical resources due to slow reallocation has resulted in deteriorated access to healthcare facilities, especially in emergency situations^14^, which are speculated to impact the risk of OHND significantly. Therefore, additional endpoints and new analyses of these topics will be required in the next phase of our research.

In a hot environment, the risk of OHND is higher than that of OHCA. In a comparison of the current study results with those on the relationship between OHCA and temperature in the pre-COVID-19 time in Japan^5^, the risk of OHND in the cold environment showed a similar relative risk as that of OHCA but in the hot environment showed a higher relative risk than that of OHCA. This might be because the OHND in this study included non-transported cases, which are not included in OHCA. In hot environments, postmortem degeneration progresses rapidly in out-of-hospital events from cardiac arrest to death. Thus, the number of cases wherein the EMS team decides not to resuscitate or transport the victim is expected to increase. Therefore, the number of cases of non-transportation may increase in the higher temperature range than at approximately 25 °C, which is calculated as MMT, and the relative risk of OHND may be evaluated more highly due to the number of non-transportation cases. In the future, considering the risk of out-of-hospital death in hot environments, the number of non-transportation cases should be included in the study to avoid underestimation.

Our study had a few limitations. First, although this study aimed to assess the risk of OHND, cases wherein the cause of death was determined at the transporting hospital could not be included in the analysis. In addition, cases of suspected criminal death in police investigations could not be included in the analysis because they were not examined by the ME, even if they were ultimately determined to be the internal cause of death as a result of the investigation. Second, there might be unmeasured confounding factors that influenced the risk of OHND, associated with the timing of epidemic waves or mass vaccination unrelated to temperature, in the post-COVID-19 period. Third, although we discussed changes in the structure of the population in Osaka City, there might be other unmeasured structural changes in the population that influenced the risk of OHND. Fourth, although a lag effect of approximately several days in the impact of ambient temperature on mortality has been reported^1,5^, our investigation was limited to weekly summary data because the goal of this study was to model the weekly average temperature and the number of OHND occurrences as simply as possible and to obtain sound evidence. However, modeling daily data may provide more insights into our findings. Although such investigations should be sensitive to modeling assumptions, it is necessary to attempt careful model-building to obtain a more precise understanding of the mechanisms behind our findings. Fifth, our investigation of the impact of the COVID-19 pandemic on mortality is limited to an overall comparison of mortality between the pre- and post-COVID-19 periods and has not reached any mechanistic explanations behind the association we observed. Recently, some studies have attempted to understand the association between changes in the COVID-19 pandemic as a time-varying factor and the mortality rate^15^. Our novel finding of the statistically significant difference in the mortality rate between the pre- and post-COVID-19 periods motivates us to conduct more detailed modeling of temporal changes in the pandemic and the mortality rate and investigate their associations in our future work. As an example of a time-varying factor, the population number used for offset terms was obtained from census results conducted in 2015 and 2020, which did not track population changes during the COVID-19 pandemic period in detail. Therefore, the impact of the COVID-19 pandemic on the number of people living alone and its effect on the risk of OHND may not have been thoroughly estimated. In particular, due to the super aging society of Japan, the number of elderly and those living alone has been increasing in Osaka City, which may have a significant impact on OHND incident risk. The effect of temperature itself on OHND incident risk may be overestimated without adequate adjustment for the effects of the increase in the elderly and living-alone populations. We would like to make this a subject of further research after continuously collecting demographic data during the COVID-19 pandemic.

In conclusion, the risk of OHND increased in both hot and cold environments, with a clear trend among the elderly and living-alone groups. Changes in the risk of OHND in the post-COVID-19 times were observed to be larger in the hot environment, with a clear increasing trend among the elderly and living with a housemate groups.

## Methods

Review of unnatural deaths and the ME system (Supplementary Fig. S2 online).

“Natural deaths” of persons are certified by their attendant doctors when they die of the diseases they had been diagnosed with. “Unnatural deaths” are generally defined as the deaths excluded from the “natural deaths.” In the Japanese medical system, almost all unnatural deaths are first recognized as OHCA or deaths outside the hospital, and EMS are always requested. Next, the arriving EMS personnel determine whether the victim is resuscitative; if unresuscitative, that is, already dead, the case is automatically reported to the police as an unnatural death. Patients determined to be resuscitative are resuscitated and transported to the hospital. When the return of spontaneous circulation is not ultimately achieved after transportation, the patient is confirmed dead in the hospital. Cases of confirmed deaths, except for those definitively diagnosed as death from disease, that is, natural death, are considered unnatural deaths and are reported to the police. In this process, unnatural deaths include cases wherein the cause of death is unknown or deaths occur from external causes, such as accidents, suicides, and suspected criminals. The reported unnatural deaths undergo police investigation in the first step to exclude crime-related deaths and death with a third-party contribution. When cases are suspected to be criminal, they undergo an autopsy at the departments of forensic medicine in the universities and are excluded from the ME’s examination. Osaka Prefecture established the Osaka Prefectural Office of ME (OPOME) as a ME system based on the Autopsy Preservation Act in Japan. MEs of the OPOME investigate the unnatural deaths that occurred in Osaka City, for which the above process has been followed. MEs estimate the cause of death and submit death certificates based on a police background check, medical history, and information on the death scene, as well as postmortem computed tomography (CT) scan, laboratory tests, and pathological examinations. In Osaka City, the number of elderly and those living alone is increasing with the super aging society of Japan, and thus the investigation of OHND by the ME system is of increasing public health importance.

### Study design and subjects

We performed a retrospective observational study using the OPOME data. Data from January 1, 2018, to December 31, 2022, were used in this study. From all cases during the study period, we extracted those of natural death based on the cause-of-death classification determined by the ME in charge. Furthermore, to eliminate the effects of potential accidental deaths, bathing-related deaths were excluded. The boundary of the COVID-19 pandemic was set on April 1, 2020, the start of the first declaration of a state of emergency in Japan. The date of death cannot be precisely determined in many unwitnessed cases, which are often included in ME cases. Accordingly, we adopted the total weekly number of cases rather than the daily number of cases. Since the weekly totals were used for the number of cases, the data on temperature were accordingly calculated as the weekly mean temperatures and used in this analysis. We obtained data on the temperature in Osaka City from the database of the Japan Meteorological Agency (https://www.data.jma.go.jp/obd/stats/etrn/index.php).

### Statistics

We applied the Poisson regression model to the outcome of the number of OHND per week.

The objective of the Poisson regression analysis was.

i. To understand the relationship between OHND and temperature and evaluate whether the risk of OHND was higher in hot or cold environments.
ii. To address how the risk of OHND changed during the COVID-19 pandemic
iii. To determine whether the COVID-19 pandemic had a more serious impact on OHND in hot or cold environments.

We defined the borderline temperature for a cold environment as the first quartile temperature obtained in this study. The first quartile temperature is consistent with the previous definition of the cutoff used for the range of moderate temperature^2^. The borderline temperature for the hot environment was set to 28 °C, which is the 86th percentile temperature observed in this study because the temperature at which the risk of mortality increases in a hot environment is reported to be the 86th percentile value of the observed temperature range in Japan^2^.

To be more specific, we applied the following Poisson regression model;

$${\text{Log E}}\left( {\text{Y}} \right) = \upalpha +\upbeta _{{1}} \times {\text{T}} +\upbeta _{{2}} \times {\text{T}}^{{2}} +\upgamma \times {\text{I}}_{{{\text{COVID}}}} +\updelta _{{1}} \times \left( {{\text{T}} - {28}} \right)_{ + } +\updelta _{{2}} \times {\text{I}}_{{{\text{COVID}}}} \times \left( {{\text{T}} - {28}} \right)_{ + } +\upeta _{{1}} \times \left( {{1}0 - {\text{T}}} \right)_{ + } +\upeta _{{2}} \times {\text{I}}_{{{\text{COVID}}}} \times \left( {{1}0 - {\text{T}}} \right)_{ + }$$

where Y and E(Y) are the observed and expected numbers of cases with OHND in each week, respectively. The variables on the right-hand side were defined as follows: T was the average temperature within a week in degrees Celsius. I_COVID_ was the dummy variable, which was 0 and 1 if the week was in the pre- and post-COVID-19 periods, respectively.

(T-28)_+_ was the linear-truncation spline function with the knot at 28 °C; (T-28)_+_  = T-28 if T >  = 28 and = 0 otherwise. Similarly, (10-T)_+_ was defined as 10-T if T < 10 and 0 otherwise.

The first three terms on the right-hand side of the model described the overall association between the average temperature and the weekly number of OHND. To capture the potential non-linear relationship, which was suggested by Kang et al.^7^, we added the quadratic term for T. The fourth term, γ × I_COVID_ describes the overall difference in the average number of OHND between the pre- and post-COVID-19 periods. The remaining terms were used to evaluate how high the risk of OHND was in hot and cold environments and how serious the impact of the COVID-19 pandemic on the risk of OHND was in hot or cold environments. The regression coefficient δ_2_ can be used to evaluate whether COVID-19 has a more serious impact in a hot environment. Parameter η_2_ played a similar role in the cold environment. Although, as mentioned, a non-linear relationship was suggested between the temperature and the OHND, we employed the linear-truncation spline function to address the hot and cold environment-specific issues, expecting the linear model to capture the local relationship and simplicity of interpretation.

### Sensitivity analysis

A study on the potential adaptation to a changing climate has reported an increase in the temperature of minimum morbidity (MMT) in recent years owing to modified susceptibility to temperature, reaching approximately 30 °C in Japan^16^. Therefore, the sensitivity analysis was performed with a borderline temperature of 30 °C for the hot environment.

Misspecification of the variance structure in the Poisson regression was a concern since it cannot capture over- or under-dispersion. We also applied the negative binomial regression model to confirm whether the results with the Poisson regression were sensitive to the variance specification.

Poisson and negative binomial regressions were applied using the glm and glm.nb functions in R.

### Subgroup analysis

Previous studies have reported an increased risk of OHCA in the elderly and those living alone^7,11^. In Osaka City, Japan, as described above, there many elderly people and many of those are living alone, and the impact on OHND incidence among this population was assumed; therefore, we performed a subgroup analysis stratified by age and living style.

Age was stratified into elderly and non-elderly at approximately 65 years of age, and lifestyle was stratified into those living alone and those living with a housemate. Based on the format of the document used to receive information from the police at the OPOME, the living-alone group was defined as those who lived in their own homes, excluding hospitals and welfare facilities, and who had no one living with them. Furthermore, we performed a sensitivity analysis using the criterion for the elderly being 75 years old.

In the model, the target population for each stratum was added as offset terms in addition to the previous variables. Although the overall population of Osaka City changed little before and after the COVID-19 pandemic period, the population of the subgroups stratified into the elderly and those living alone were expected to change. Therefore, we used offset terms to avoid inaccurate risk assessment by considering this study's absolute number of OHND.

Poisson regression model with offset term;

$${\text{Log E}}\left( {\text{Y}} \right) = {\text{Log }}\left( {{\text{offset}}_{{{\text{subgroup}}}} } \right) + \upalpha +\upbeta _{{1}} \times {\text{T}} +\upbeta _{{2}} \times {\text{T}}^{{2}} +\upgamma \times {\text{I}}_{{{\text{COVID}}}} +\updelta _{{1}} \times \left( {{\text{T}} - {28}} \right)_{ + } +\updelta _{{2}} \times {\text{I}}_{{{\text{COVID}}}} \times \left( {{\text{T}} - {28}} \right)_{ + } +\upeta _{{1}} \times \left( {{1}0 - {\text{T}}} \right)_{ + } +\upeta _{{2}} \times {\text{I}}_{{{\text{COVID}}}} \times \left( {{1}0 - {\text{T}}} \right)_{ + }$$

where the offset_subgroup_ is the number of population subgroups considered, such as the elderly and those living alone. The model is expressed as follows;

$${\text{Log }}\left\{ {{\text{E}}\left( {\text{Y}} \right)/{\text{offset}}_{{{\text{subgroup}}}} } \right\} = \upalpha +\upbeta _{{1}} \times {\text{T}} +\upbeta _{{2}} \times {\text{T}}^{{2}} +\upgamma \times {\text{I}}_{{{\text{COVID}}}} +\updelta _{{1}} \times \left( {{\text{T}} - {28}} \right)_{ + } +\updelta _{{2}} \times {\text{I}}_{{{\text{COVID}}}} \times \left( {{\text{T}} - {28}} \right)_{ + } +\upeta _{{1}} \times \left( {{1}0 - {\text{T}}} \right)_{ + } +\upeta _{{2}} \times {\text{I}}_{{{\text{COVID}}}} \times \left( {{1}0 - {\text{T}}} \right)_{ + }$$

By investigating the regression parameters of the model, we can address the impact of explanatory variables on the ratio of the absolute number of OHND to the target population.

We obtained the data of the stratified population from the National Census in Japan, with results available to the public. Pre-COVID-19 pandemic data were obtained from the census in 2015, and post-COVID-19 pandemic data were obtained in 2020.

In 2015, there were 668,698 elderly, 1,978,096 non-elderly, 657,205 living alone, and 2,033,980 living-with-housemate individuals. In 2020, there were 676,821 elderly, 1,977,406 non-elderly, 784,785 living alone, and 1,967,627 living-with-housemate individuals.

### Ethics declarations and consent to participate

The Ethical Review Board Osaka University Hospital, the institutional ethics committee of the University of Osaka Graduate School of Medicine, approved this study according to the Ethical Guidelines for Medical and Health Research Involving Human Subjects (Approval No. 20418). The Committee did not require informed consent for the use of data that did not contain personal information. All methods were carried out in accordance with the relevant guidelines and regulations.

### Supplementary Information

Supplementary Figures.

Supplementary Tables.

## Data Availability

This published article and its supplementary information files include all data generated or analyzed during this study.

## References

[1] Anderson BG, Bell ML (2009). Weather-related mortality: How heat, cold, and heat waves affect mortality in the United States. Epidemiology.

[2] Gasparrini A (2015). Mortality risks attributable to high and low ambient temperature: A multicountry observational study. Lancet.

[3] Gerber Y, Jacobsen SJ, Killian JM, Weston SA, Roger VL (2006). Seasonality and daily weather conditions in relation to myocardial infarction and sudden cardiac death in Olmsted County, Minnesota, 1979 to 2002. J. Am. Coll. Cardiol..

[4] Chen T, Sarnat SE, Grundstein AJ, Winquist A, Chang HH (2017). Time-series analysis of heat waves and emergency department visits in Atlanta, 1993 to 2012. Environ. Health Perspect..

[5] Onozuka D, Hagihara A (2017). Extreme temperature and out-of-hospital cardiac arrest in Japan: A nationwide, retrospective, observational study. Sci. Total Environ..

[6] Onozuka D, Hagihara A (2017). Out-of-hospital cardiac arrest risk attributable to temperature in Japan. Sci. Rep..

[7] Kang SH (2016). Heat, heat waves, and out-of-hospital cardiac arrest. Int. J. Cardiol..

[8] Lai PH (2020). Characteristics associated with out-of-hospital cardiac arrests and resuscitations during the novel coronavirus disease 2019 pandemic in New York City. JAMA Cardiol..

[9] Hubert H, Baert V, Beuscart JB, Chazard E (2020). Use of out-of-hospital cardiac arrest registries to assess COVID-19 home mortality. BMC Med. Res. Methodol..

[10] Marijon E (2020). Out-of-hospital cardiac arrest during the COVID-19 pandemic in Paris, France: A population-based, observational study. Lancet Public Health.

[11] Ong ME (2011). Spatial variation and geographic-demographic determinants of out-of-hospital cardiac arrests in the city-state of Singapore. Ann. Emerg. Med..

[12] Baldi E (2020). Out-of-hospital cardiac arrest during the Covid-19 outbreak in Italy. N. Engl. J. Med..

[13] Pranata R, Lim MA, Yonas E, Siswanto BB, Meyer M (2020). Out-of-hospital cardiac arrest prognosis during the COVID-19 pandemic. Intern. Emerg. Med..

[14] Nakahara S, Inada H, Ichikawa M, Tomio J (2021). Japan’s slow response to improve access to inpatient care for COVID-19 patients. Front Public Health.

[15] Onozuka D (2022). Reduced mortality during the COVID-19 outbreak in Japan, 2020: A two-stage interrupted time-series design. Int. J. Epidemiol..

[16] Vicedo-Cabrera AM (2018). A multi-country analysis on potential adaptive mechanisms to cold and heat in a changing climate. Environ. Int..

