# Supplementary material for: Association of atmospheric temperature with out-of-hospital natural deaths occurrence before and during the COVID-19 pandemic in Osaka, Japan
Source: Sci Rep. 2023 Oct 28;13:18529. doi: 10.1038/s41598-023-45816-7 (PMC10613267; doi:10.1038/s41598-023-45816-7)
Supplement: Supplementary file 1 — Supplementary Figures. [file 41598_2023_45816_MOESM1_ESM.pptx]

## Slide 1
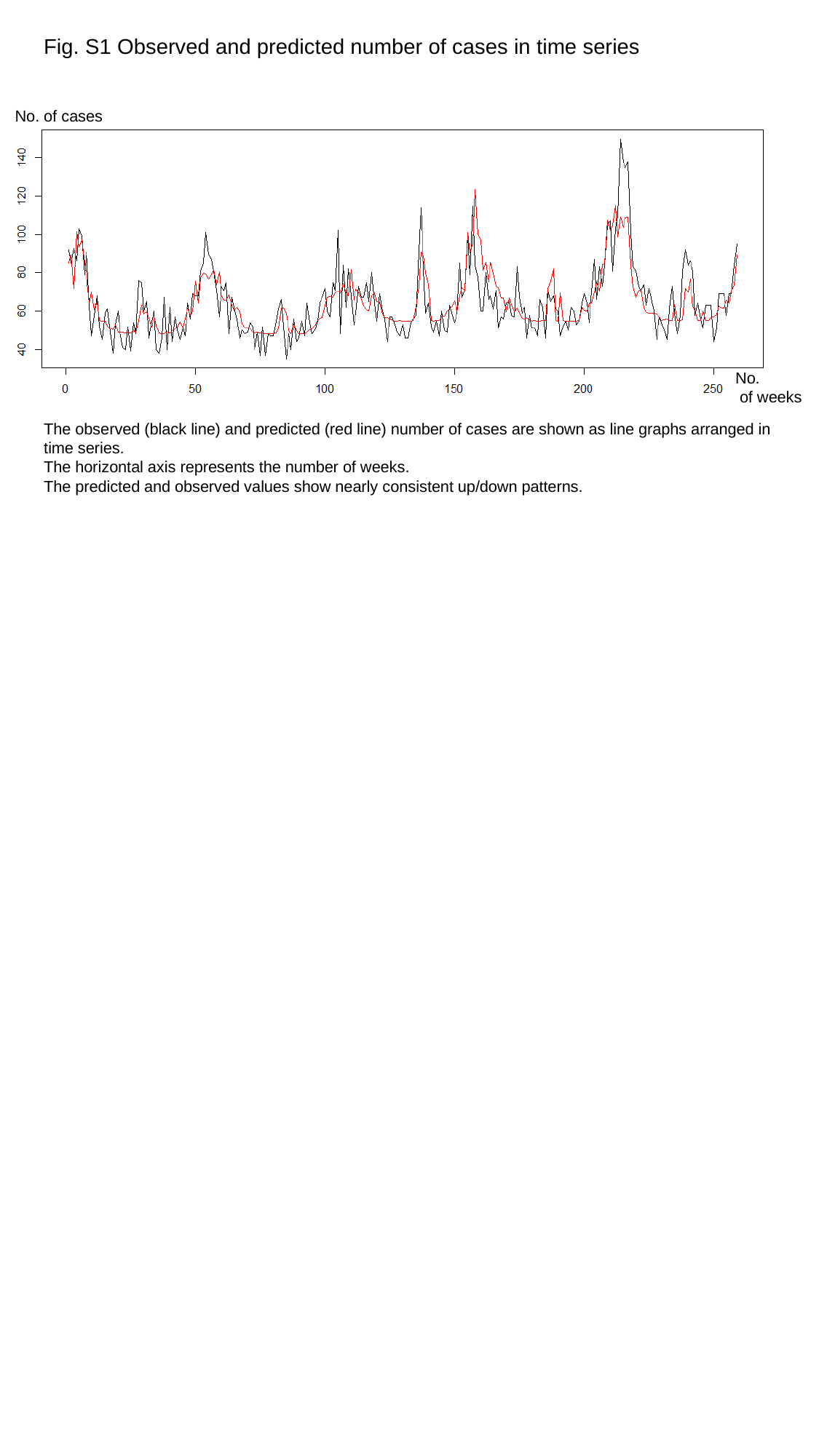

Fig. S1 Observed and predicted number of cases in time series
No. of cases
No.
 of weeks
The observed (black line) and predicted (red line) number of cases are shown as line graphs arranged in time series.
The horizontal axis represents the number of weeks.
The predicted and observed values show nearly consistent up/down patterns.

## Slide 2
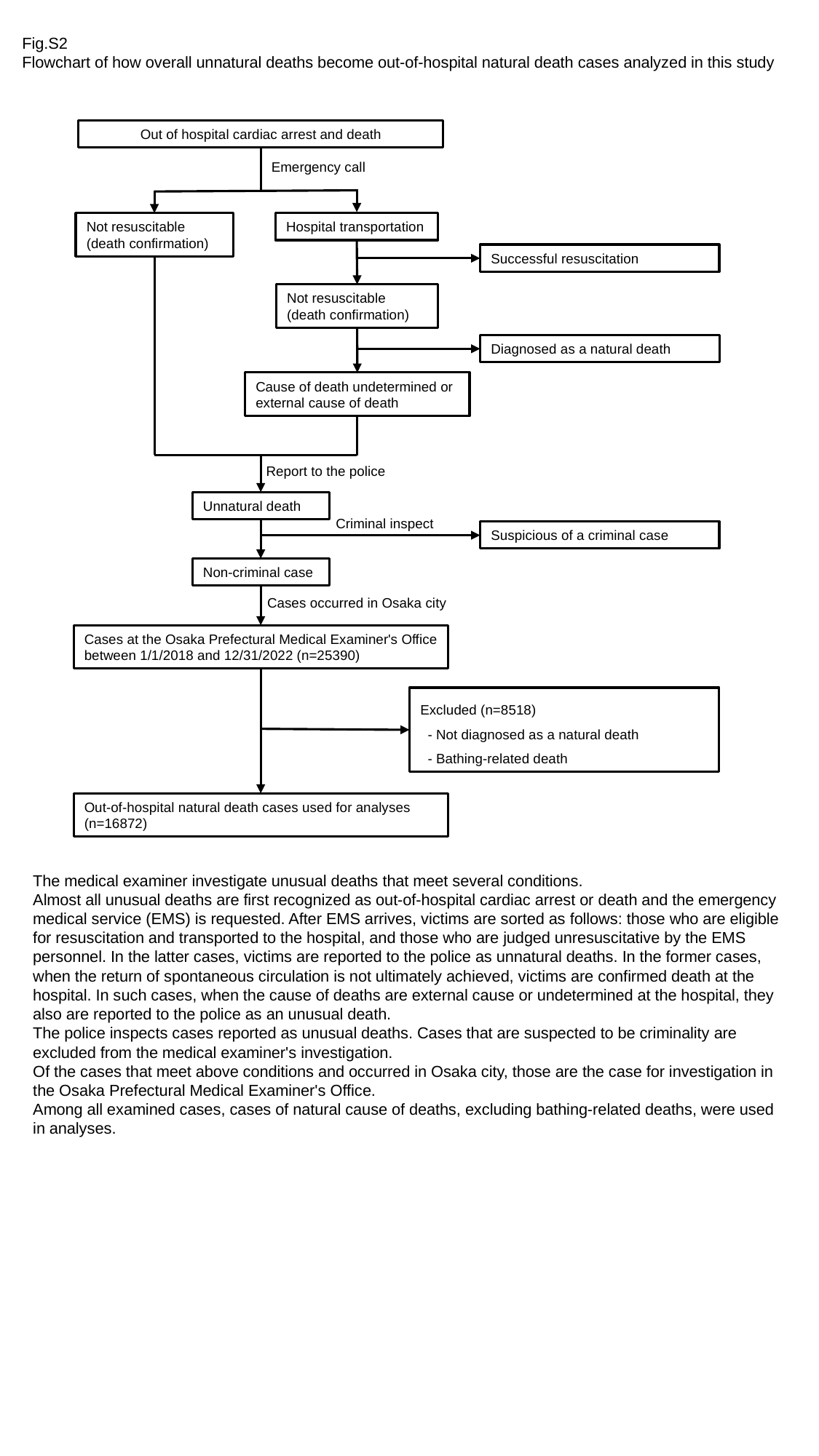

Fig.S2
Flowchart of how overall unnatural deaths become out-of-hospital natural death cases analyzed in this study
Out of hospital cardiac arrest and death
Emergency call
Not resuscitable (death confirmation)
Hospital transportation
Successful resuscitation
Not resuscitable (death confirmation)
Diagnosed as a natural death
Cause of death undetermined or external cause of death
Unnatural death
Criminal inspect
Suspicious of a criminal case
Non-criminal case
Report to the police
Cases occurred in Osaka city
Cases at the Osaka Prefectural Medical Examiner's Office between 1/1/2018 and 12/31/2022 (n=25390)
Excluded (n=8518)
 - Not diagnosed as a natural death
 - Bathing-related death
Out-of-hospital natural death cases used for analyses (n=16872)
The medical examiner investigate unusual deaths that meet several conditions.
Almost all unusual deaths are first recognized as out-of-hospital cardiac arrest or death and the emergency medical service (EMS) is requested. After EMS arrives, victims are sorted as follows: those who are eligible for resuscitation and transported to the hospital, and those who are judged unresuscitative by the EMS personnel. In the latter cases, victims are reported to the police as unnatural deaths. In the former cases, when the return of spontaneous circulation is not ultimately achieved, victims are confirmed death at the hospital. In such cases, when the cause of deaths are external cause or undetermined at the hospital, they also are reported to the police as an unusual death.
The police inspects cases reported as unusual deaths. Cases that are suspected to be criminality are excluded from the medical examiner's investigation.
Of the cases that meet above conditions and occurred in Osaka city, those are the case for investigation in the Osaka Prefectural Medical Examiner's Office.
Among all examined cases, cases of natural cause of deaths, excluding bathing-related deaths, were used in analyses.
