# Supplementary material for: Association of atmospheric temperature with out-of-hospital natural deaths occurrence before and during the COVID-19 pandemic in Osaka, Japan
Source: Sci Rep. 2023 Oct 28;13:18529. doi: 10.1038/s41598-023-45816-7 (PMC10613267; doi:10.1038/s41598-023-45816-7)
Supplement: Supplementary file 2 — Supplementary Tables. [file 41598_2023_45816_MOESM2_ESM.docx]

Table.S1 Sensitive analysis with a borderline temperature of 30°C for the hot environment

|  |  |  | RR (95% CI) | p-value |
| --- | --- | --- | --- | --- |
| Overall relationship | | with T: exp (β_1_) | 0.9148 (0.8957-0.9342) | <0.001 |
|  |  | with T^2^: exp (β_2_) | 1.0020 (1.0014-1.0025) | <0.001 |
| Relative risk of post- vs pre-COVID-19 period: exp (γ) | | | 1.1465 (1.1053-1.1893) | <0.001 |
| Common effects of  pre- and post-COVID-19 period | Relative risk per 1°C in hot environment (T>30°C): exp (δ_1_) | | 1.0408 (0.9640-1.1237) | 0.306 |
|  | Relative risk per 1°C in cold environment (T<10°C): exp (η_1_) | | 0.9925 (0.9693-1.0162) | 0.529 |
| Impacts of  COVID-19 pandemic on | Relative risk per 1°C in hot environment (T>30°C): exp (δ_2_) | | 1.1315 (1.0400-1.2310) | 0.004 |
|  | Relative risk per 1°C in cold environment (T<10°C): exp (η_2_) | | 1.0219 (1.0036-1.0406) | 0.019 |

The minimum mortality temperature (MMT) has reported to be rising in recent years due to modified susceptibility to temperature. Thus, sensitive analysis was performed with a borderline temperature of 30°C for the hot environment, with similar results.

Table.S2 Sensitive analysis with a negative binomial distribution as error structure

|  |  |  | RR (95% CI) | p-value |
| --- | --- | --- | --- | --- |
| Overall relationship | | with T: exp (β_1_) | 0.9334 (0.9035-0.9644) | <0.001 |
|  |  | with T^2^: exp (β_2_) | 1.0014 (1.0005-1.0022) | 0.002 |
| Relative risk of post- vs pre-COVID-19 period: exp (γ) | | | 1.1410 (1.0866-1.1982) | <0.001 |
| Common effects of  pre- and post-COVID-19 period | Relative risk per 1°C in hot environment (T>28°C): exp (δ_1_) | | 1.0496 (0.9972-1.1048) | 0.064 |
|  | Relative risk per 1°C in cold environment (T<10°C): exp (η_1_) | | 1.0069 (0.9726-1.0423) | 0.698 |
| Impacts of  COVID-19 pandemic on | Relative risk per 1°C in hot environment (T>28°C): exp (δ_2_) | | 1.0543 (1.0063-1.1046) | 0.026 |
|  | Relative risk per 1°C in cold environment (T<10°C): exp (η_2_) | | 1.0226 (0.994-1.0495) | 0.092 |

To validate the misspecification of the variance structure, the negative binominal regression model was applied, with similar results.

Table.S3 Subgroup analysis stratified by age and living style

|  |  |  | age | | | | lifestyle | | | |
| --- | --- | --- | --- | --- | --- | --- | --- | --- | --- | --- |
|  |  |  | non-elderly | | elderly | | living with housemate | | living alone | |
|  |  |  | RR (95% CI) | p-value | RR (95% CI) | p-value | RR (95% CI) | p-value | RR (95% CI) | p-value |
| Overall relationship | with T: exp (β_1_) | | 0.9687 (0.9196-1.0203) | 0.230 | 0.9189 (0.8929-0.9457) | <0.001 | 0.9513 (0.9160-0.9879) | 0.010 | 0.9140 (0.8838-0.9453) | <0.001 |
|  | with T^2^: exp (β_2_) | | 1.0004 (0.9991-1.0018) | 0.535 | 1.0017 (1.0009-1.0025) | <0.001 | 1.0009 (0.9999-1.0019) | 0.084 | 1.0018 (1.0009-1.0027) | <0.001 |
| Relative risk of post- vs pre-COVID-19 period: exp (γ) | | | 1.0792 (0.9985-1.1665) | 0.055 | 1.1565 (1.1074-1.2078) | <0.001 | 1.2659 (1.1954-1.3405) | <0.001 | 0.9142 (0.8691-0.9616) | 0.001 |
| Common effects of  pre- and post-COVID-19 period | Relative risk per 1°C in hot environment (T>28°C): exp (δ_1_) | | 1.0585 (0.9747-1.1495) | 0.176 | 1.0761 (1.0289-1.1254) | 0.001 | 1.0272 (0.9655-1.0929) | 0.396 | 1.1008 (1.0459-1.1587) | 0.000 |
|  | Relative risk per 1°C in cold environment (T<10°C): exp (η_1_) | | 1.0202 (0.9666-1.0767) | 0.468 | 1.0003 (0.9716-1.0298) | 0.986 | 1.0335 (0.9942-1.0743) | 0.096 | 0.9828 (0.9499-1.0169) | 0.319 |
| Impacts of  COVID-19 pandemic on | Relative risk per 1°C in hot environment (T>28°C): exp (δ_2_) | | 1.0288 (0.9568-1.1063) | 0.443 | 1.0403 (1.0014-1.0807) | 0.042 | 1.0947 (1.0378-1.1548) | 0.001 | 1.0028 (0.9598-1.0477) | 0.900 |
|  | Relative risk per 1°C in cold environment (T<10°C): exp (η_2_) | | 0.9839 (0.9446-1.0247) | 0.433 | 1.0155 (0.9947-1.0367) | 0.145 | 1.0084 (0.9809-1.0366) | 0.554 | 1.0119 (0.9872-1.0372) | 0.348 |

CI indicates the confidence interval, RR the rate ratio.

Subgroup analyses were performed with factors of age and living style that have been suggested to be associated with OHCA in previous studies. Age was stratified into elderly and non-elderly at approximately 65 years of age, and lifestyle was stratified into those living alone and those living with a housemate. In the model, the target population for each stratum was added as offset terms in addition to the previous variables.

In the non-elderly group, all regression coefficients show no significance.

In the elderly group, the relative risk in post- vs. pre-COVID-19 period is significant, and the increase in relative risk per 1°C from pre- to post-COVID-19 period was significant only in the hot environment (T>28°C).

In the living with housemate group, the relative risk in post- vs. pre-COVID-19 period was significant and the largest among all subgroups, and the increase in relative risk per 1°C was significant only in the hot environment (T>28°C).

In the living-alone group, the relative risk in post- vs. pre-COVID-19 period is significantly lower than 1, and the increase in relative risk per 1°C was insignificant in the both hot and cold environment.

In the cold environment (T<10°C), the change of relative risk per 1°C from pre- to post-COVID-19 period was insignificant in all subgroups.

Table.S4 Sensitivity analysis for subgroup analysis focusing on a borderline age of 75 years in the elderly population

|  |  |  | age | | | |
| --- | --- | --- | --- | --- | --- | --- |
|  |  |  | non-elderly | | elderly | |
|  |  |  | RR (95% CI) | p-value | RR (95% CI) | p-value |
| Overall relationship | with T: exp (β_1_) | | 0.9501 (0.9159-0.9856) | 0.005 | 0.9117 (0.8797-0.9450) | <0.001 |
|  | with T^2^: exp (β_2_) | | 1.0009 (0.9999-1.0019) | 0.064 | 1.0019 (1.0009-1.0028) | <0.001 |
| Relative risk of post- vs pre-COVID-19 period: exp (γ) | | | 1.0560 (0.9996-1.1157) | 0.047 | 1.1204 (1.0609-1.1832) | <0.001 |
| Common effects of  pre- and post-COVID-19 period | Relative risk per 1°C in hot environment (T>28°C): exp (δ_1_) | | 1.0678 (1.0083-1.1308) | 0.022 | 1.0769 (1.0180-1.1393) | 0.008 |
|  | Relative risk per 1°C in cold environment (T<10°C): exp (η_1_) | | 1.0107 (0.9734-1.0495) | 0.570 | 1.0001 (0.9645-1.0370) | 0.997 |
| Impacts of COVID-19 pandemic on | Relative risk per 1°C in hot environment (T>28°C): exp (δ_2_) | | 1.0250 (0.9750-1.0775) | 0.324 | 1.0473 (0.9986-1.0983) | 0.052 |
|  | Relative risk per 1°C in cold environment (T<10°C): exp (η_2_) | | 1.0000 (0.9725-1.0282) | 0.998 | 1.0142 (0.9886-1.0404) | 0.271 |

Recently, the age of 75 years has become another criterion for identifying the elderly. Thus, a sensitivity analysis was performed with a borderline age of 75 years for the elderly, with similar results.
